# Effect of Arginine Vasopressin on Intraoperative Hypotension Caused by Oral Administration of 5-Aminolevulinic Acid

**DOI:** 10.1155/2023/1745373

**Published:** 2023-05-06

**Authors:** Gen Owada, Hideo Nishizawa, Yuki Matoyama, Eri Watanabe, Keigo Mitsuda, Naoki Kaneko, Yasuhiro Kimura, Taikan Nanao, Junichi Fujimoto

**Affiliations:** ^1^Department of Intensive Care Medicine, Yokohama Rosai Hospital, Yokohama, Japan; ^2^Department of Anesthesia, Yokohama Rosai Hospital, Yokohama, Japan

## Abstract

5-Aminolevulinic acid (5-ALA) is used for the photodynamic diagnosis of malignant tumors and has been effectively utilized to improve the complete resection rate and reduce the risk of tumor recurrence. However, intraoperative hypotension is a common adverse effect of oral 5-ALA, and it occasionally progresses to severe prolonged hypotension requiring high-dose catecholamine administration. We report a case of intraoperative hypotension due to oral 5-ALA in which arginine vasopressin (AVP) administration was effective for increasing the blood pressure. A 77-year-old man scheduled for a craniotomy for glioma was administered 5-ALA orally before surgery. After the induction of anesthesia, his blood pressure decreased substantially. Although we administered various vasopressor agents, hypotension was prolonged. However, after starting a continuous administration of AVP, the systolic blood pressure increased, and the hemodynamic parameters remained stable during the remainder of the operation. 5-ALA administration may lower blood pressure by inducing nitric oxide production, and AVP inhibits inducible nitric oxide synthase messenger RNA expression and interleukin-1*β*-stimulated nitric oxide production. In light of these mechanisms, AVP may be a reasonable treatment agent for hypotension induced by 5-ALA.

## 1. Introduction

5-Aminolevulinic acid (5-ALA) is used for the photodynamic diagnosis of malignant tumors [1]. When 5-ALA is taken orally before surgery, it is metabolized to protoporphyrin IX (PPIX), which accumulates in cancer cells and emits red fluorescence when irradiated with blue light, thus aiding in tumor visualization [1]. Recently, fluorescent cystoscopy using photodynamic agents has been effectively utilized for improving the complete resection rate and reducing the risk of tumor recurrence [1].

5-ALA-induced intraoperative hypotension is a common side effect [2], and there are several reports on the occurrence of severe prolonged hypotension requiring high-dose catecholamine [3, 4]. The mechanism of hypotension has been reported in several ways [5]. Recently, the production of nitric oxide (NO) in vascular endothelial cells has been suggested as a possible mechanism of hypotension [6]. If NO production is a key mediator of 5-ALA-induced hypotension, arginine vasopressin (AVP) could be an effective treatment for 5-ALA-induced intraoperative hypotension although we know of no reports of this mechanism.

Herein, we report a case of intraoperative hypotension due to oral 5-ALA where AVP administration effectively increased blood pressure.

## 2. Case Presentation

A 77-year-old man with the American Society of Anesthesiologists physical status II, a body weight of 72 kg, and a body mass index of 28.5 was scheduled for craniotomy of a glioma. He had a history of well-controlled hypertension (blood pressure around 120/80 mmHg), stage II chronic kidney disease, and coronary artery disease. Preoperative examination results such as chest radiography, laboratory tests, and physical examination were all normal. He was taking benidipine hydrochloride 4 mg twice daily, bisoprolol fumarate 2.5 mg once daily, and enalapril maleate 2.5 mg once daily for hypertension. He only took bisoprolol fumarate on the morning of the operation. He had fasted for 12 hours before surgery. His blood pressure was 133/85 mmHg on the operation day, and aminolevulinic acid hydrochloride (1.5 g, ALABEL; Nobelfarma Co., Ltd., Tokyo, Japan) was administered orally 2 hours before surgery. Before anesthesia induction, his blood pressure and heart rate were 100/55 mmHg and 80 beats/min, respectively (Fig available here). Anesthesia was induced with fentanyl 100 mcg, propofol 90 mg, and rocuronium 40 mg and maintained with desflurane 3% and remifentanil 0.05–0.2 mcg/kg/min. We did not use the BIS monitor to guide the depth of sedation. The lowest blood pressure after anesthesia induction was 55/30 mmHg. There were no anaphylactic symptoms such as a rash, edema, or wheezing. Despite acute volume resuscitation and the total administration of 0.8 mg of phenylephrine and 24 mg of ephedrine, the hypotension (blood pressure around 70/40 mmHg) lasted over 20 minutes. The bolus and continuous administration of norepinephrine (NE) up to 0.1 mcg/kg/min were also not effective. However, after starting continuous administration of AVP at 0.03 unit/min, the systolic blood pressure rose over 90 mmHg and the hemodynamic status remained stable throughout the operation. The surgical procedure was uneventful and lasted 4 hours 19 minutes. His fluid intake was 4250 ml, urine output was 600 ml, amount of bleeding was 81 ml, and hemoglobin concentration was 12.9–13.7 g/dL during the surgery. He was transferred to the intensive care unit (ICU) after extubation to monitor the hemodynamic status. After ICU admission, the patient was immediately weaned off AVP and his blood pressure was stable until ICU discharge the next day. In the general ward, his blood pressure was around 120/80 mmHg. He was discharged on postoperative day 8.

## 3. Discussion

The use of oral 5-ALA for the photodynamic diagnosis of malignant cells during surgery has been associated with an increased gross total resection rate [7] and a reduced risk of recurrence compared with the traditional operative procedure [8]. In particular, it has been widely used for transurethral bladder tumor resection (TURBT) and craniotomy for glioma [7, 8]. However, intraoperative hypotension is a common side effect of 5-ALA [9–11]. Several cases of severe and catecholamine-resistant hypotension have been reported [6, 9, 12]. One retrospective study showed that the prevalence of postinduction hypotension after 5-ALA administration was 70% in TURBT and craniotomy [3]. The same study found that various vasopressor agents, including ephedrine, phenylephrine, dopamine, NE, and epinephrine, reduced the minimum mean blood pressure to 49 mmHg; however, AVP was not used. Only one case report mentioned the use of AVP for intraoperative hypotension induced by 5-ALA. Up to 0.08 unit/min of AVP was continually administered to achieve stable hemodynamics [4]. However, that report did not document the effectiveness of AVP for hemodynamic stability. In addition, there are no reports on the management of intraoperative hypotension due to oral 5-ALA administration.

Our patient also experienced severe and catecholamine-resistant hypotension. We consider that the hypotension was not associated with his medicines, benidipine hydrochloride and enalapril maleate, because he did not take them on the morning of the operation. Besides, the pharmacologic half-lives of these drugs are not so long; the half-life of benidipine hydrochloride is 2.3 ± 0.7 hours and enalapril maleate is 8.6 ± 3.6 hours. We also consider that hypotension was not associated with fasting, depth of anesthesia, and pharmacological half-life. The fasting duration was usual for this operation and the hypotension continued after volume resuscitation. Although we did not use the BIS monitor, the anesthesia was not deep in terms of the degree of desflurane and remifentanil. The half-life of 5-ALA is 2.27 hours and PPIX generation from 5-ALA which induces to activate soluble guanylate cyclase, relaxes the smooth muscle of arteries, and results in hypotension is 4.91 hours [5]. Because the operation duration was 4.33 hours, the effect of PPIX could be still active at ICU admission. In terms of that, hemodynamic stability at ICU admission could be due to awakening from anesthesia. However, that hypotension especially during the operation was severe prolonged requiring high-dose catecholamine administration. After starting continuous administration of AVP, the systolic blood pressure rose steadily and we got hemodynamic stability; thus we consider AVP was most effective in stabilizing hemodynamics for intraoperative hypotension induced by 5-ALA.

Although the precise mechanisms of intraoperative hypotension following 5-ALA administration are unknown, its association with NO production in vascular endothelial cells was recently reported. Morisawa et al. showed [6] that NO production increased after 5-ALA administration in a concentration-dependent manner. They suggested that 5-ALA may lower blood pressure by increasing the NO production. In contrast, AVP is a well-known peptide hormone that induces vasoconstriction via the V1 receptor on vascular smooth muscle cells [13]. Regarding vasoconstriction, AVP binding to the V1 receptor activates phospholipase C, resulting in the production of inositol 1,4,5-triphosphate (InsP3). InsP3 induces calcium ion (Ca^2+^) release from the sarcoplasmic reticulum and increases intracellular Ca^2+^ concentration, thus causing vasoconstriction and vasopressor effects [13]. Additionally, AVP inhibits inducible NO synthase messenger RNA expression via the V1 receptor and interleukin-1*β*-stimulated NO production in cultured rat vascular smooth muscle cells [14, 15]. This mechanism of inhibiting NO production may explain why AVP is an effective vasopressor for intraoperative hypotension induced by 5-ALA. In other words, oral 5-ALA induces intraoperative hypotension because of NO production, and AVP administration inhibits the NO production to stabilize the hemodynamics. The *α*-agonists such as phenylephrine and NE also introduce vasoconstriction by increasing intracellular Ca^2+^ concentration. However, from the standpoint of NO production, this mechanism may not be effective for 5-ALA-induced intraoperative hypotension whereas AVP administration may be effective.

## 4. Conclusion

5-ALA-induced intraoperative hypotension can be severe and prolonged at times, requiring a high dose of catecholamine. When faced with that situation, we consider using AVP for hemodynamic stability. AVP is pharmacologically reasonable and may be more effective than other vasopressor agents.

## Data Availability

The data used to support the findings of the study are included within the article.

## Supplementary Materials

Supplementary Materials Figure: anesthesia chart. It showed refractory and prolonged hypotension despite the administration of several vasopressor agents. Even with catecholamine administration, it was less effective. However, arginine vasopressin administration was effective in increasing the blood pressure. After continuous arginine vasopressin administration, we got hemodynamics stability.
