# Supplementary figures and images for: Effect of Arginine Vasopressin on Intraoperative Hypotension Caused by Oral Administration of 5-Aminolevulinic Acid
Source: Case Rep Anesthesiol. 2023 May 6;2023:1745373. doi: 10.1155/2023/1745373 (PMC10182879; doi:10.1155/2023/1745373)

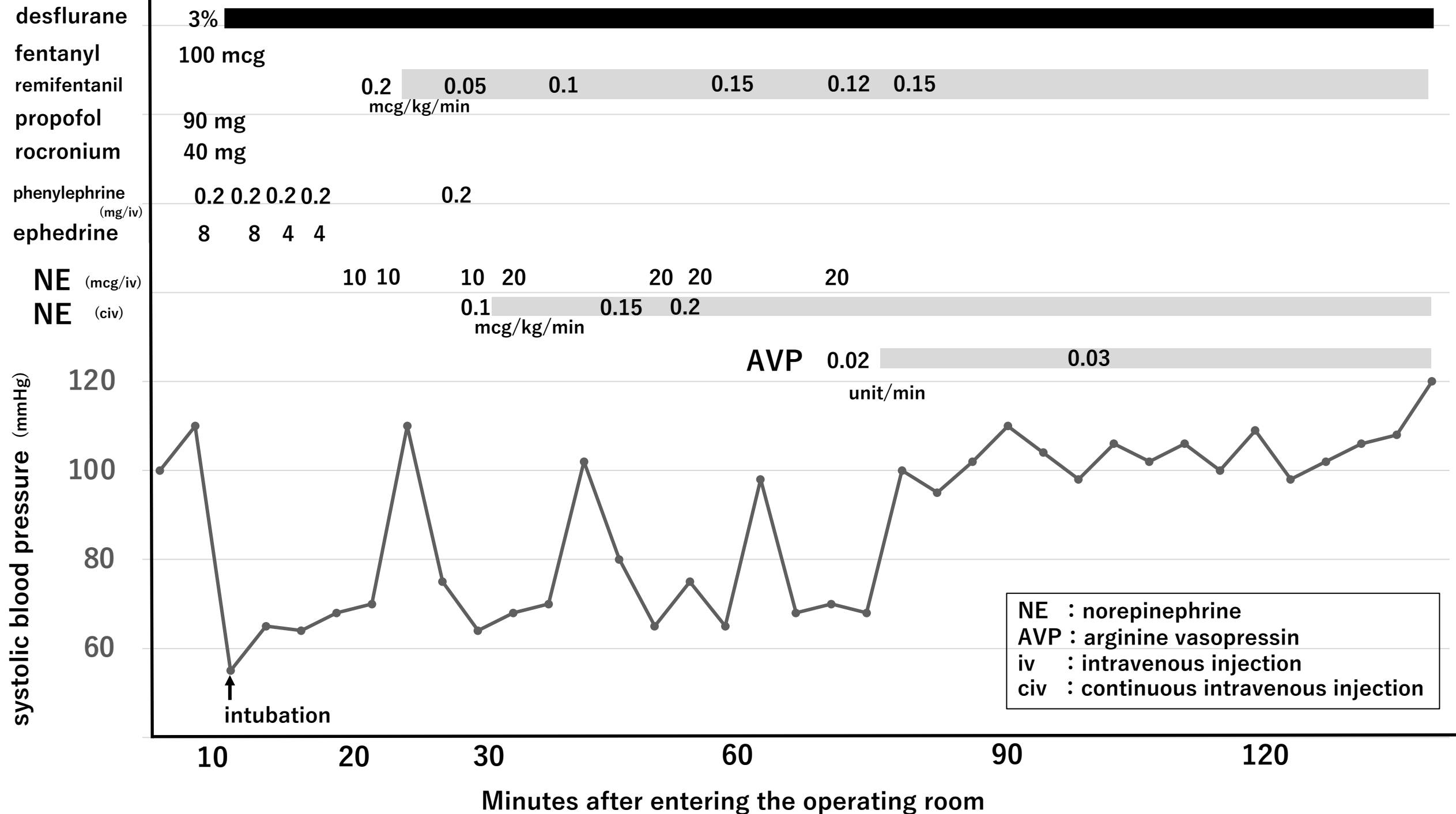

Supplement: Supplementary Materials — Figure: anesthesia chart. It showed refractory and prolonged hypotension despite the administration of several vasopressor agents. Even with catecholamine administration, it was less effective. However, arginine vasopressin administration was effective in increasing the blood pressure. After continuous arginine vasopressin administration, we got hemodynamics stability. [file 1745373.f1.pdf]
